# Toward DNA-Based Recording of Biological Processes

**DOI:** 10.3390/ijms25179233

**Published:** 2024-08-26

**Authors:** Hyeri Jang, Sung Sun Yim

**Affiliations:** 1 Department of Biological Sciences, Korea Advanced Institute of Science and Technology (KAIST), Daejeon 34141, Republic of Korea; 2 Graduate School of Engineering Biology, Korea Advanced Institute of Science and Technology (KAIST), Daejeon 34141, Republic of Korea; 3 KAIST Institute for BioCentury, Korea Advanced Institute of Science and Technology (KAIST), Daejeon 34141, Republic of Korea; 4 Korea Research Institute of Bioscience and Biotechnology (KRIBB), Daejeon 34141, Republic of Korea

**Keywords:** DNA-based data storage, molecular recording, cellular memory, synthetic biology

## Abstract

Exploiting the inherent compatibility of DNA-based data storage with living cells, various cellular recording approaches have been developed for recording and retrieving biologically relevant signals in otherwise inaccessible locations, such as inside the body. This review provides an overview of the current state of engineered cellular memory systems, highlighting their design principles, advantages, and limitations. We examine various technologies, including CRISPR-Cas systems, recombinases, retrons, and DNA methylation, that enable these recording systems. Additionally, we discuss potential strategies for improving recording accuracy, scalability, and durability to address current limitations in the field. This emerging modality of biological measurement will be key to gaining novel insights into diverse biological processes and fostering the development of various biotechnological applications, from environmental sensing to disease monitoring and beyond.

## 1. Introduction

Biological processes are inherently complex and dynamic. Living organisms interact with each other and their environments by generating diverse biomolecules and metabolites, and these interactions continuously change over time. For example, microbial cells in the gut microbiome constantly sense environmental changes and respond by regulating the expression of specific genes necessary for their survival [1]. In multicellular organisms, the intricate regulation of numerous genes controls the differentiation of multiple cell types throughout development [2]. However, many of these dynamics remain poorly understood since native biological environments are often inaccessible, and tracking multiple biological events over time is still challenging [3].

**Figure 1:**
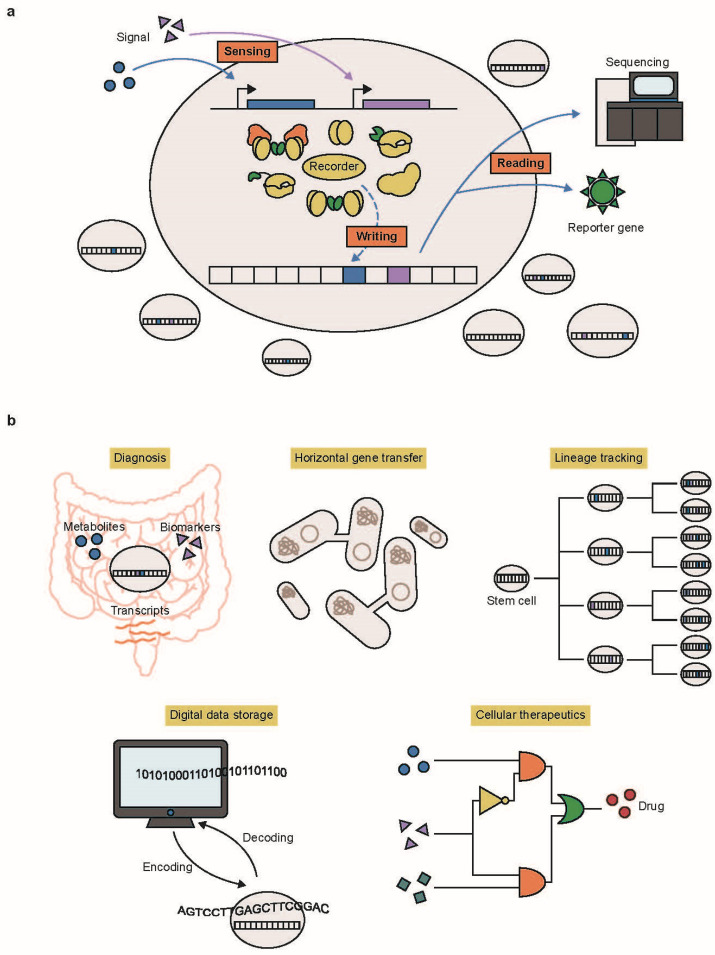
(**a**) In DNA-based cellular recording, various environmental or cellular signals activate molecular recorders. Once activated, these recorders alter the DNA sequence or epigenetic states to store the data. The recorded data can be retrieved through sequencing or reporter gene expression. (**b**) Examples of DNA-based cellular recording applications include diagnosing cellular states, understanding horizontal gene transfer (HGT) events within the microbiome, tracking cellular lineages, storing digital data, and constructing genetic circuits for therapeutic purposes.

While several approaches such as temporal RNA-seq [4,5,6] and biosensors [7,8,9] have been devised to address these challenges in biological measurement, they are still constrained by their temporal resolution and the number of channels available for data acquisition. Utilizing DNA as a data storage medium provides high-capacity storage, high density, and long-term stability to encode various types of data [10,11]. Advanced next-generation sequencing (NGS) technologies have facilitated convenient, cost-effective, and high-throughput decoding of information stored in DNA [12]. Furthermore, the inherent compatibility between DNA data storage and living systems has spurred the development of various DNA-based cellular recording techniques, which have the potential to acquire multiple and temporal biological information without disrupting cells (Figure 1a) [3]. Many different applications of DNA-based cellular recording have been demonstrated, such as diagnosing disease biomarkers [13,14], capturing horizontal gene transfer (HGT) events [15,16], tracking cellular lineages throughout embryonic development [17,18], storing digital data [19,20], and constructing genetic circuits for therapeutic applications [21] (Figure 1b).

In this review, we explore the principles of genome editing-based cellular recording systems, highlighting their benefits and applications across various fields. We also examine potential strategies to overcome current limitations in this area. This emerging method of biological measurement is crucial for obtaining new insights into diverse biological processes and advancing various biotechnological applications.

## 2. Recombination-Based Cellular Recording

**Figure 2:**
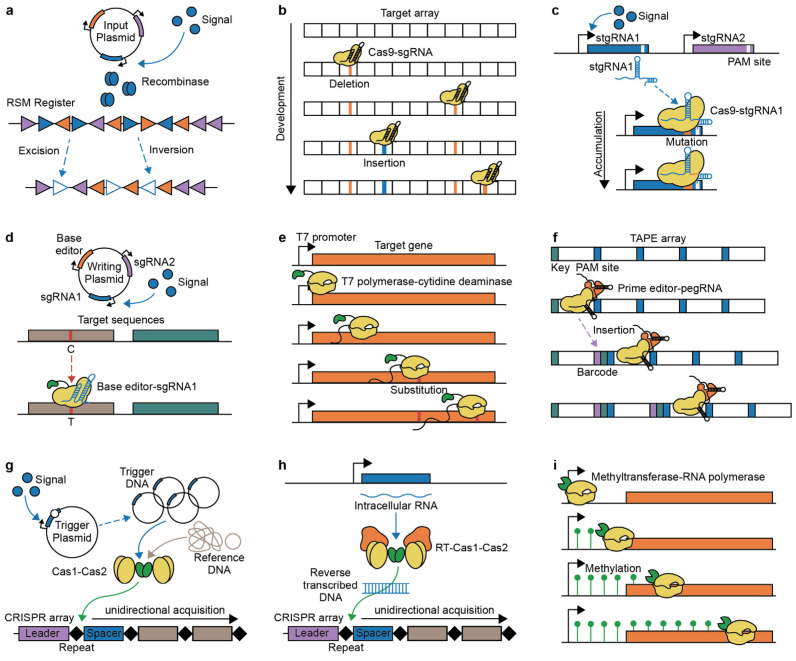
The principles of DNA-based recording systems are illustrated. (**a**) Recombinase-based state machines (RSMs): Orthogonal recombinases are activated in response to multiple signals. Depending on the order of signals, recombinases facilitate either excision or inversion of the RSM register, enabling the recording of the temporal order of multiple signals. (**b**) Genome editing of synthetic target arrays for lineage tracing (GESTALT): A contiguous array of target barcodes is edited by Cas9 nuclease-sgRNA throughout cell development. The accumulated patterns of deletions and insertions enable the reconstruction of lineage tree. (**c**) Mammalian synthetic cellular recorders integrating biological events (mSCRIBE): Multiple self-targeting guide RNAs (stgRNAs) and Cas9 nucleases are used to edit the stgRNA gene itself for monitoring biological signals. Within the cell population, self-targeting patterns correlate with either the duration or intensity of the signals. (**d**) CRISPR-mediated analog multi-event recording apparatus (CAMERA): Inducible base editors and sgRNAs generate C∙G to T∙A point mutations at recording sites. The editing frequencies depend on signal amplitude or duration, and the editing patterns indicate the order of events. (**e**) T7 polymerase-driven continuous editing system: T7 polymerase fused to cytidine deaminase transcribes a specific gene downstream, continuously generating substitution patterns. (**f**) DNA Typewriter: The pegRNA, consisting of key sequences, barcodes, and type guide sequences, is expressed under a promoter. The prime editor inserts the key and barcode sequences adjacent to the PAM site in a unidirectional manner, enabling temporal recording within cells. (**g**) Temporal recording in arrays by CRISPR expansion (TRACE): Biological signals activate replication proteins, facilitating the replication of the pTrig plasmid. The Cas1–Cas2 complex integrates trigger DNA into the CRISPR array at a higher frequency compared to reference sequences. The unidirectionality of CRISPR acquisition allows for the temporal recording of multiple signals. (**h**) Record-seq: Expressed intracellular RNA is reverse transcribed into DNA sequences by RT. The resulting double-stranded DNA is then integrated into the CRISPR array by the Cas1–Cas2 complex. This system enables transcriptome-scale recording. (**i**) DCM-time machine (DCM-TM): The fusion protein of DCM methyltransferase and RNA polymerase is activated by an inducible signal. When the RNA polymerase acts on genes and active enhancers, DCM methyltransferase marks the methylation patterns along the sequences.

Recombinases are enzymes that mediate site-specific recombination by catalyzing excision, inversion, and integration of specific target DNA sequences, depending on the orientation of flanking homologous regions. These site-specific DNA recombinases have been utilized to construct various genetic circuits for cellular recording, such as permanent genetic memories and reversible genetic switches, which can be analyzed by recombination site sequences or reporter gene expression [22,23,24,25,26,27]. For the development of the recombination-based genetic memory system with >1-byte capacity, Yang et al. bioinformatically identified orthogonal phage integrases with their cognate recognition (attB-attP) sites and constructed a ‘memory array’ by linearly concatenating the recognition sites for each integrase [23]. With the orthogonal recombinase–recombination site pairs, the recording of temporally ordered signals could also be demonstrated as ‘recombinase-based state machines’ (RSMs) (Figure 2a) [28]. In the RSM concept, sequence states could be generated on DNA registers (memory arrays) made up of overlapping and orthogonal recombinase recognition sites. Depending on the order of a set of chemical inputs, corresponding recombination events could result in expected sequence states of the two-input five-state and three-input 16-state registers and cellular behaviors.

Beyond recording the occurrence (presence or absence) of events, the recombinase-based approach could also encode the duration and intensity of biological events. The ‘synthetic cellular recorders integrating biological events’ (SCRIBE) system was developed to record analog information, such as the magnitude and time course of inputs, within living cell populations by converting transcriptional signals into the production of single-stranded DNA (ssDNA), followed by ssDNA-based genome editing [29]. In the SCRIBE system, retrons, composed of a non-coding RNA (ncRNA) region with multicopy single-stranded RNA (msr) and multicopy single-stranded DNA (msd), as well as retron reverse transcriptase (retron RT) [30], are utilized to produce the ssDNAs [31,32,33]. And the ssDNAs write information at specific genomic loci as recombination frequencies within cell populations when single-strand annealing proteins (SSAPs) are co-expressed. Recently, the recombination efficiency of ssDNA retrons was improved by knocking out or knocking down cellular ssDNA-specific exonucleases, which affect the intracellular stability of ssDNA, enabling a broader range of applications for the system [16].

While recombinase-based recording systems have primarily been established within model bacterial systems, their implementations have been successfully demonstrated in non-model bacteria and even in eukaryotes, including human and plant cells [34,35,36]. However, scalability remains challenging due to the limited number of available orthogonal recombinases. To address this, computational mining of efficient and orthogonal recombinases from microbial genomes could further expand the recombinase toolbox [37]. Alternatively, exploiting recombinases with orthogonal attachment sites and synthetic transcription factors together could increase memory capacity for each recombinase and enable much faster recombination [38].

## 3. Implementation of Genome Editing for Molecular Recording

Genome editing involves the precise alteration of genomic sequences in living organisms by generating targeted insertions, deletions, and substitutions. While various genome-editing techniques, such as zinc-finger nucleases (ZFN) and transcription activator-like effector nuclease (TALEN), have demonstrated potential for effective genome engineering [39,40], the emergence of CRISPR technology has facilitated programmable genome engineering, leading to the development of diverse DNA-based recording systems [41,42,43].

### 3.1. CRISPR-Cas9 Barcoding-Based Lineage Tracing

The CRISPR-Cas9 system, a prokaryotic adaptive immune system, is composed of the Cas9 nuclease and single-guide RNA (sgRNA). CRISPR-Cas9 is a robust technology that facilitates genome engineering, screening, and transcription regulation by precisely recognizing and cleaving specific locations and editing target sequences within the genome [44,45,46,47,48]. The CRISPR-Cas9 nuclease causes DNA double-stranded breaks (DSBs) at specific locations, leading to irreversible insertions or deletions during the repair processes. The accumulation of these mutations could be utilized as unique barcodes for individual cells or cellular events in DNA-based cellular recording.

CRISPR-barcoding has been utilized for cellular recording, especially lineage tracing, by accumulating mutations such as deletions and insertions during cell division. For example, the ‘genome editing of synthetic target arrays for lineage tracing’ (GESTALT) strategy demonstrated this potential by applying CRISPR-Cas9 barcodes to fertilized zebrafish (*Danio rerio*) eggs for cumulative lineage barcoding (Figure 2b) [17]. Their lineage-informative barcodes were deciphered through DNA sequencing, allowing for the elucidation of lineage relationships based on mutation patterns. Similarly, the ‘memory by engineered mutagenesis with optical in situ readout’ (MEMOIR) system generates an irreversible collapse of a set of barcoded scratchpads by Cas9 targeted to the scratchpads during cell proliferation, enabling the recording of gene expression dynamics [49,50]. The states of these collapsed scratchpads were identified through multiplex single-molecule RNA fluorescence hybridization (smFISH) using sequential barcoding to multiplex different mRNAs by sequential hybridization [51].

To further improve CRISPR-Cas9 barcoding-based lineage tracing, combining CRISPR-Cas9 barcoding with single-cell RNA sequencing (scRNA-seq) allows for the acquisition of cellular transcriptomes and cell-type identification, facilitating robust lineage tracing of embryonic development [52,53,54,55] and tumor evolution [56]. While CRISPR-Cas9 barcoding is an effective method for cellular lineage tracing, the activity of the Cas9 nuclease can result in an off-target effect. Furthermore, scalability is restricted by the number of target arrays or barcodes, limiting its applications to early developmental processes [17].

### 3.2. Applications of Self-Targeting gRNA

Self-targeting CRISPR, also known as homing CRISPR, is a modified CRISPR-Cas9 system where the Cas9-gRNA complex directs its activity to the gRNA locus itself [57]. As self-targeting guide RNA (stgRNA) or homing guide RNA (hgRNA) contains a protospacer-adjacent motif (PAM) directly recognized by the Cas9 nuclease, it provides both guiding ability and target sites. When the stgRNA barcoding elements detect their target sequences to trigger mutations, the diversity of stgRNAs can be generated for barcoding and lineage tracing purposes [18]. While canonical CRISPR-Cas9 barcoding approaches capture only specific trajectories or moments due to their dependency on barcode sequences, stgRNA approaches establish a more independent barcoding system and produce substantially diverse barcodes.

The stgRNA approaches have shown potential for mapping cell development. For barcoding and recording cell lineages in mice, the Mouse for Actively Recording Cells 1 (MARC1) line carried multiple stgRNAs in its genome sequences and was crossed with Cas9 knock-in mouse [18,58]. In their offspring, the activation of stgRNAs generated diverse mutation patterns, which were passed to daughter cells with additional mutations. This MARC1 system could construct a stable mouse line for barcoding and minimize the unwanted loss of patterns from large deletions. Additionally, self-targeting CRISPR approaches have been utilized to record biological events. For example, the ‘mammalian synthetic cellular recorders integrating biological events’ (mSCRIBE) system accumulates mutations in their stgRNA containing PAM sequences by linking the expression of stgRNA or Cas9 to specific biological events (Figure 2c) [59]. The frequency of accumulated stgRNA mutations within cell populations is correlated with the duration or magnitude of the biological signals. Moreover, beyond the relative duration of signals, the elapsed time of biological signals could also be gauged using stgRNAs that decay the intact target sequence frequency [60]. Most self-targeting CRISPR approaches have provoked deletions for their marking but face the risk of erasing existing records. Instead, terminal deoxynucleotidyl transferase (TdT) has been introduced to add new DNA sequences, thereby avoiding progressive erasure [13]. However, the increased lengths of stgRNA mediated by the insertions could also decrease editing efficiencies, limiting the scalability of the systems.

### 3.3. Base Editing-Based Cellular Recording

Deletions or insertions formed through the DSB repair pathway, including non-homologous end joining (NHEJ) or homologous recombination (HR) [61], may lead to cellular toxicity and the risk of overwriting new barcodes in existing recordings. Base editing, a CRISPR-based genome editing technique, differs from others by not relying on Cas9 nuclease, instead employing dead Cas9 (dCas9) or nickase Cas9 (nCas9). Both lose the ability to cleave double-stranded DNA, reducing cellular toxicity but retaining the ability to bind target sequences guided by gRNAs. These modified Cas9 nucleases have been fused with base editors such as cytidine deaminase or adenine deaminase to modulate point mutations [62,63,64].

CRISPR-based base editing has facilitated the cellular recording of extracellular signals, especially effective for long-term analog recording due to its substantial storage capacity. Base editing-based recording has been demonstrated in both bacteria and mammalian cells. For example, in the ‘CRISPR-mediated analog multi-event recording apparatus’ (CAMERA) system, engineered bacteria demonstrated their recording ability in response to various stimuli, such as chemical signals, viral infections, and light exposure, by activating multiple gRNAs in response to these stimuli (Figure 2d) [65]. Simultaneously, the system was applied in mammalian cells, enabling the recording of chemical signals and Wnt signals. In CAMERA, expressed gRNAs direct a base editor composed of dCas9 and cytidine deaminase to targeted DNA sequences, facilitating C∙G to T∙A mutations. These mutation frequencies within populations indicate the magnitude or duration of specific signals. The sequential and temporal logics of multiple signals could also be constructed by more complex circuits [66]. In addition, dead Cas12a (dCas12a) has also been fused with a base editor for single nucleotide editing [67]. To enhance the efficiency of multiplex modulation, dCas12a was engineered through structure-guided protein engineering [68]. With an adenine base editor, it could effectively record much information in human cells [21]. With base editing-based recording approaches, analog characteristics such as the magnitude and duration of exogenous signals were reconstructed by the frequency of specific mutations at target sites within populations [21,65,66]. However, simultaneously distinguishing both remains challenging. Furthermore, most base editing approaches have focused on cellular recording at the population level. To address this, cellular recording at the single-cell level has been demonstrated through long-read sequencing of a ‘canvas’ with multiple target sites for base editing [69] or editing endogenous interspersed repeat regions for lineage tracing [70]. Additionally, recording multiple endogenous transcripts at the single-cell level could be performed by sensing transcripts with reprogrammed tracrRNAs (Rptrs) to convert the target endogenous mRNAs into gRNAs and mediate base editing to target DNA [71].

Base-editing-based approaches have also adopted other DNA-binding proteins to guide the target sequences. For example, the T7 polymerase-driven continuous editing system demonstrated that transcriptional activities under the T7 promoter can be recorded through continuous nucleotide substitution mutations by exploiting T7 RNA polymerase (T7 RNAP) fused to cytidine deaminase (Figure 2e) [72]. The T7 promoter was integrated into genomic loci of specific genes, allowing the T7 RNAP-cytidine deaminase complex to constitutively access the T7 promoter and its downstream region, facilitating transcription and sequence editing. Furthermore, base-editing is not limited to DNA; it also enables transcriptional and temporal recording in RNA by utilizing RNA-specific adenosine deaminase with an RNA-binding domain [73,74].

### 3.4. Prime Editing-Based Recording Methods

Prime editing is a genome editing technique where target DNA is replaced by new genetic sequences [75,76]. It has the advantage of excluding bystander editing and Cas-independent off-target effects, which are challenges of base editing. The prime editor, comprising nCas9 fused to reverse transcriptase (RT), induces single-stranded breaks (SSBs) at specific locations directed by prime editor guide RNA (pegRNA). The pegRNA carries an editing sequence adjacent to the binding sequence as a template for reverse transcription. Specific sequences generated by RT are encoded by the prime editor, allowing for precise editing, such as DNA substitutions, insertions, and deletions, at targeted sites without requiring DSBs or donor DNA templates

Prime editing has been employed for robust temporally resolved cellular recording by producing sequential arrays with incorporated barcodes in the edited sequences. Individual pegRNAs with unique barcodes are inserted sequentially into specific genomic loci [20,77]. The ‘prime editing cell history recording by ordered insertion’ (peCHYRON) inserts 20 bp sequences, consisting of 3 bp signature mutations as the barcode and 17 bp constant propagator sequences adjacent to the PAM site [77]. With each cycle of insertion, the previous binding sequences are inactivated by being moved away from the PAM site. Another prime editing-based approach, DNA Typewriter, accomplished sequential recording by inserting short key sequences and barcodes into a tandem array of monomers containing the PAM sequence, subsequently shifting the position of the type of guide sequence (Figure 2f) [20,78]. Exploiting this sequential barcoding in the array mediated the encoding and decoding of short text messages within cell populations, collecting diverse encoded single cells. Within single cells, 3 bp barcodes were assigned to characters among alphabets, numbers, and symbols, and the barcode position in the tandem array encoded the order in sets of four characters.

For further multiplex recording, the ‘enhancer-derived genomic recording of transcriptional activity in multiplex’ (ENGRAM) integrates multiple signals and enhancer-specific barcodes into pegRNA [79]. This allowed for the scalable insertion of specific barcodes, capturing multiple transcriptional activities simultaneously. Despite their multiplexing and order dependency, the low efficiency of prime editing-based recording remains challenging. To improve the efficiency and precision of prime editing, engineered prime editors have been developed. For example, pegRNAs were modified to include structured 3′ motif sequences that enhance RNA stability and prevent degradation, thereby increasing prime editing efficiencies [80]. Additionally, engineered RT and Cas9 nuclease were developed through phage-assisted evolution to further enhance prime editing efficiency [81]. These advancements in the prime editing approach can facilitate the incorporation of barcodes for rare events, thereby enhancing the reliability and accuracy of temporal recording.

## 4. CRISPR Adaptation for Temporal Recording

The CRISPR-Cas system functions as an adaptive immune response in prokaryotes, encompassing three main stages: adaptation or acquisition, expression and maturation, and interference. The CRISPR adaptation process involves recognizing foreign DNA sequences and integrating them into the CRISPR array to establish a genetic memory of viral infections. These CRISPR arrays consist of a leader sequence, short repeat sequences, and spacers derived from foreign DNA. These arrays are transcribed into CRISPR RNA (crRNA) and subsequently processed to facilitate interference activity. The CRISPR integrases and Cas1–Cas2 complex incorporate DNA sequences, typically ranging from 30 to 40 bp, as new spacer sequences into the CRISPR array [82,83]. The new spacer sequences are integrated at the leader end of the CRISPR array, positioning the newest spacer ahead of older spacers [84].

Unidirectional CRISPR adaptation has facilitated the temporal recording of cellular events. Arbitrary DNA sequences of a specific size can be acquired as spacers in the CRISPR arrays by expressing CRISPR integrases Cas1 and Cas2 [85,86]. Recently, methods for capturing biological events have been developed by integrating intracellular DNA sequences. For example, the ‘temporal recording in arrays by CRISPR expansion’ (TRACE) system records temporal environmental signals into the CRISPR arrays by utilizing a copy number-inducible trigger plasmid (pTrig), which contains the phage P1 lytic replication initiation protein coding gene downstream of an inducible promoter (Figure 2g) [87]. In response to environmental signals, the increase in pTrig copy number led to a higher frequency of trigger DNA acquisition in the CRISPR array compared to reference sequences such as genomic and plasmid DNA. Furthermore, in the TRACE system, multiplex recording of three environmental signals was demonstrated by using a three-barcoded sensor population. This further enabled the encoding of arbitrary digital data in the CRISPR array by electronic stimulation of the trigger plasmid, maintaining robust long-term records in living cells [19].

The complex of RT and Cas1–Cas2 has been employed to record transcriptional events through CRISPR adaptation. The Record-seq strategy showed transcriptome-scale molecular recording by leveraging RT-Cas1 and Cas2 to directly capture transcripts into the CRISPR array (Figure 2h) [88]. As the acquisition frequencies of spacers depend on the source RNA abundance, highly expressed genes were captured more frequently in the CRISPR arrays. To detect rarely acquired spacers, the ‘selective amplification of expanded CRISPR arrays’ (SENECA) method was developed to specifically amplify the acquired spacers for deep sequencing [89]. Record-seq demonstrated its ability to noninvasively assess cellular transcriptional events in the intestines of mice under different dietary or environmental conditions [14]. More recently, the Retro-Cascorder system utilized retrons, previously mentioned in the SCRIBE system, to reverse transcribe engineered ncRNA barcodes into ssDNA. Then, two generated ssDNA hybridized to form duplex DNA for CRISPR acquisition [90,91]. The expression of distinct barcoded ncRNA under different inducible promoters enabled CRISPR acquisition of different duplex sequences, mediating multiplex temporal recording.

CRISPR adaptation-based approaches are powerful for temporal information recording; however, their recording efficiencies and applicable host range remain constrained. Enhancing CRISPR adaptation efficiency by utilizing internal nucleases or evolved CRISPR integrases holds promise for expanding the recording capacity and applicability of these systems, making them more versatile and effective across diverse biological contexts. For example, Cas4 nucleases or endonucleases such as DnaQ and ExoT inherently control the size and orientation of integrated spacers via asymmetric trimming [92,93,94]. These nucleases coordinate with CRISPR integrases, facilitating efficient CRISPR adaptation. Furthermore, evolving CRISPR integrases through directed evolution and enriching the mutant integrases by perpetual DNA packaging and transduction (PeDPaT) offer the potential to improve CRISPR-adaptation-based recording [95,96].

## 5. Using DNA Methylation for Biological Recording

DNA methylation is a major epigenetic process characterized by the addition of a methyl group to nucleic acid bases, such as cytosine and adenine, without altering the original sequences. This reversible modification mediates the regulation of gene expression in development and disease [97,98]. DNA methyltransferases also play a role in the prokaryotic defense system associated with the restriction-modification (RM) system [99,100]. Three prevalent methylation patterns, including 5-methylcytosine (5mC), N4-methylcytosine (4mC), and N6-methyladenine (6mA), are controlled by their catalytic writer, reader, and eraser enzymes. Recent advances in DNA methylome mapping technologies have enabled the analysis of these methylation profiles [101,102,103].

Synthetic epigenetic circuits, especially those involving targeted DNA methylation, have regulated specific gene expression levels and durably retained cellular epigenetic memory [104,105,106,107]. While CRISPRa and CRISPRi methods transiently manipulate gene function, targeted DNA methylation can provide long-term regulations. For example, an engineered bacterial 6mA regulatory system could be utilized to record biological events and control transcriptional events in mammalian cells, since 6mA modification is not common in eukaryotes [108]. In response to environmental signals, a 6mA writer, a fusion of an engineered Dam methylase, and an engineered zinc finger for DNA binding mediated targeted methylation at GATC motifs to construct epigenetic memory, recording the presence of environmental signals [109].

Genome-wide transcriptome recording could also be demonstrated using the DCM-time machine (DCM-TM) system through epigenome editing (Figure 2i) [110]. This system analyzed methylation patterns by methylated DNA sequencing (MeD-seq) based on LpnPI digestion of DCM methylated position [111]. An inducible fusion protein of DCM methyltransferase and the RNA polymerase 2 subunit b labeled methylation patterns on transcribed genes and active enhancers when the gene was transcribed by RNA polymerase. This strategy was utilized to understand the genetic activity and temporal dynamics of intestinal stem cells (ISCs) during their differentiation into enterocytes. DNA methylation-based approaches could further increase their utility by using methyltransferase and demethylase for reversible epigenetic modification, as demonstrated in the CRISPRoff and CRISPRon systems [112].

While methylation-based recording approaches offer extensive scalability for recording transcriptomes by using the whole genome sequence as a recording site, they still have certain limitations. Notably, methylation-based techniques for recording the temporal order of various signals and analog characteristics have not been demonstrated. Additionally, the requirement for specific recognition sites for each methyltransferase may limit their applications.

## 6. Outlook and Discussion

DNA-based cellular recordings using DNA recombination, CRISPR systems, and DNA methylation have enabled the generation of permanent memories of environmental and biological events in living cells. In this review, we examined various DNA-based cellular recording systems, focusing on their principles, advantages, and limitations (Table 1). Unlike existing reviews on cellular recording [3,113,114,115,116], we covered the most recent cellular recording techniques, such as prime editing-based multiplexed temporal recording systems. Additionally, we introduced methylation-based cellular recording strategies alongside the commonly discussed recombinase, CRISPR nuclease, and CRISPR integrase systems.

Molecular recording of cellular events can be applied to diagnosing cellular states, capturing HGT events, tracking cell lineage, storing digital data in DNA, and developing cellular therapeutics. Selecting an appropriate recording system will be necessary for specific applications because each strategy has different advantages and scalability. For instance, the CRISPR-Cas spacer acquisition strategy possesses a distinctive ability to record horizontal gene transfer (HGT) across a cell population by directly capturing mobile DNA from complex environments [15]. When combined with genetic logic computation or sophisticated computational algorithms, DNA-based cellular recording approaches have the potential to mediate the control of cellular functions based on cellular memory [117] and to reconstruct cellular lineages [118].

We anticipate that improving DNA-based cellular recorders by enhancing their sensitivity, scalability, and durability will be key to utilizing molecular recording across various applications. While the sensitivity of most molecular recorders is limited to an hour or day scale, it is important to address stimuli that occur on a second or minute scale for responding to instant signals. Developing methods to increase the sensitivity for cellular recording at such high temporal resolution will provide real-time monitoring capabilities, which are essential for applications such as detecting rapid changes in cellular states or environmental conditions. A recent study with minute resolution demonstrated the potential for highly sensitive encoding of environmental signals [119]. Engineered TdT transduced these signals by incorporating specific nucleotides in response to cation concentrations, such as Co^2+^, Ca^2+^, and Zn^2+^, and temperature changes within 1 min in vitro. Similar to this engineered TdT, exploiting highly sensitive enzymes could improve the resolution of cellular recording.

Furthermore, expanding the capability for multiplexing and temporal recording will be necessary as these characteristics tend to be inversely proportional. Encoding temporal information of two or three environmental signals is relatively straightforward; however, managing temporal transcriptional recording on a genome-wide scale is still challenging and requires significant experimental and computational advancements. Expanded scalability to support long-term genome-wide transcriptional recording with high temporal resolution will allow for comprehensive monitoring of complex biological processes and interactions over time. A potential approach to enhance these capabilities is to combine the multiplexed and quantitative recording capacity of ENGRAM with the sequential recording capacity of DNA Typewriter [20,79]. The pegRNAs linked to signal-responsive cis-regulatory elements (CREs) that are targeted to a tandem array of partial target sites could potentially mediate unidirectional insertions of barcodes for temporal recording by shifting the editable positions.

Finally, enhancing the durability and robustness of recorded data by minimizing off-target effects and ensuring long-term stability will provide reliable data for extended studies and applications, such as longitudinal tracking of cellular changes. The off-target effects can occur not only in approaches using CRISPR-Cas nucleases but also in those using CRISPR adaptation machineries [120]. To overcome these challenges, better control of CRISPR off-target effects and the integration of robust memory maintenance mechanisms will be essential. To minimize off-target effects, engineered sgRNAs can increase the specificity of CRISPR activity by varying the hairpin structure of sgRNAs [121] or by delivering off-target-directed short gRNA while maintaining on-target efficiencies [122]. Furthermore, engineered enzymes can reduce the risks of off-target effects [123]. We expect this emerging DNA-based modality of biological measurement will be key to gaining novel insights into diverse biological processes and fostering the development of various biotechnological applications, from environmental sensing to disease monitoring and beyond.

## Figures and Tables

**Table 1:** Summary of major DNA-based cellular recording systems.

System	Approach	Information Type	Sensitivity (Timescales of Cellular Recording)	Scalability	Durability	Temporal Information	Storage Place	Features	Citation
RSM	Recombination of DNA register	Chemical	Hour scale (Fast)	Medium	Short	Yes	Plasmid	Applied to build state-dependent gene regulation programs	[28]
SCRIBE	Recombination of retron RT-DNA into genomic DNA	Chemical, Light	Day scale (Slow)	Medium	Long	No	Genome	Encoding of analog memory, reversible system	[29]
GESTALT	CRISPR-Cas9 targeted to synthetic target arrays	Cell differentiation	Day scale (Slow)	Low	Long	Yes	Genome	Mapping cell lineage information, vulnerable to off-target effect	[17]
mSCRIBE	CRISPR-Cas9 and self-targeting guide RNA targeted itself	Inflammation, Chemical	Day scale (Slow)	Medium	Long	Yes	Genome	Encoding of analog memory, vulnerable to off-target effect	[59]
CAMERA	Cas9 nuclease or base editor targeted to recording site	Chemical, Phage infection, Light, Cellular state	Hour scale (Fast)	Medium	Long	Yes	Plasmid, Genome	Encoding of analog memory, reversible system, universal system between bacteria and mammalian cells	[65]
HyperCas12a base editor system	Cas12a base editor targeted to recording circuit	Chemical, Cellular state	Hour scale (Fast)	Medium	Long	No	Plasmid, Genome	Encoding of analog memory, applied to sense-and-respond circuits	[21]
T7 polymerase-driven base editing	Base editor fused to T7 RNA polymerase targeted to T7 promoter-controlled gene sequence	Transcription	Hour scale (Fast)	Low	Long	No	Plasmid, Genome	Accompanied continuous mutagenesis to target site	[72]
DNA Typewriter	Sequential prime editng of target sequences	Transfection, Cell differentiation	Day scale (Slow)	High	Long	Yes	Genome	Applied to record complex event histories and short digital data	[20]
ENGRAM	Prime editor programmed to insert CRE-specific barcode sequence	Enhancer activity, Cellular state	Day scale (Slow)	High	Long	Yes	Genome	Could be coupled with DNA Typewriter system for temporal recording	[79]
TRACE	CRISPR adaptation of copy inducible plasmid into CRISPR array	Chemical	Hour scale (Fast)	Medium	Long	Yes	Genome	Applied to record temporal biological/digital data	[87]
Record-seq	RT-Cas1 and Cas2-based acquisition of RNA transcripts into CRISPR array	Transcription	Hour scale (Fast)	High	Long	Yes	Genome	Genome-wide transcriptional information	[88]
Retro-Cascorder	CRISPR adaptation of retron RT-DNA into CRISPR array	Chemical	Hour scale (Fast)	Medium	Long	Yes	Genome	Identification of molecular events and their orders in individual cells	[90]
DCM-TM	Methyltransferase DCM fused to RNA polymerase targeted to transcript	Transcription, Enhancer activity	Day scale (Slow)	High	Long	No	Genome	Applied to track cellular state in mouse intestine	[110]

## Data Availability

Not applicable.
